# The frailty mortality link in people with chronic diseases pertaining to 13 body organ systems: findings from the UK Biobank study

**DOI:** 10.1007/s11357-025-01980-4

**Published:** 2025-12-06

**Authors:** Justine Dastouet, Aurore Fayosse, Louis Jacob, Archana Singh-Manoux, Séverine Sabia, Benjamin Landré

**Affiliations:** 1 Epidemiology of Ageing and Neurodegenerative Diseases, Université Paris Cité, INSERM U1153, CRESS, 10 Avenue de Verdun, Paris, 75010 France; 2 Department of Physical Medicine and Rehabilitation, Lariboisière-Fernand Widal Hospital, Université Paris Cité, AP-HP Paris, France; 3 Research and Development Unit, Parc Sanitari Sant Joan de Déu, CIBERSAM, ISCIII, Dr. Antoni Pujadas, 42, Sant Boi de Llobregat, Barcelona, Spain; 4 UCL Brain Sciences, University College London, London, UK

**Keywords:** Ageing, Frailty, Chronic disease, Multimorbidity, Mortality, UK Biobank

## Abstract

Frailty in older adults, particularly those with chronic diseases, has a robust association with risk of mortality, but whether this is the case in middle-aged adults is unclear. We examined the frailty-mortality association in middle-aged adults with chronic diseases and multimorbidity. Data on Fried’s frailty phenotype (robust, prefrail, frail) and 47 chronic diseases, mapped to 13 body organ systems, was available on 230,960 participants of the UK Biobank study (mean age 57.8, range 38.0–72.0). The mortality follow-up was 13.3 (± 2.1) years, and associations with mortality were examined using Cox regression, adjusted for socio-demographic factors and health behaviours. Compared to the robust group, frailty (HR, 2.32 (95% confidence intervals, 2.21; 2.43)) and prefrailty (1.35 (1.31; 1.39)) in those with diseases in any body organ system had a higher risk of mortality. Frailty was associated with a higher mortality risk for all 13 groups (all *p* < 0.01); estimates ranged from 2.25 (2.12; 2.39) for circulatory system diseases to 2.97 (2.60; 3.39) for the eye system. The same was the case for prefrailty; estimates ranged from 1.33 (1.23; 1.43) to 1.61 (1.03; 2.51). Between 19 and 34% of the mortality risk in the 13 disease groups could potentially be explained by frailty/prefrailty. Frailty (*p* < 0.01) and prefrailty (*p* < 0.01) had stronger associations with mortality in those with diseases affecting multiple organ systems. These findings highlight the importance of frailty in middle-aged adults with chronic diseases, suggesting that a third of the excess risk of mortality could potentially be addressed by improving frailty status.

**Supplementary information:**

The online version contains supplementary material available at https://doi.org/10.1007/s11357-025-01980-4.

## Introduction

Frailty describes a state characterised by decline across multiple physiological systems, increased susceptibility to stressors, and a higher risk of adverse outcomes such as falls, disability, and mortality [1]. Several tools have been developed to measure frailty, and much of the research in this domain [2] is based on Fried’s Frailty (FFP) [3]. The FFP can be used in diverse settings and has the advantage of being a disease-agnostic tool.

Measures of frailty are increasingly used in individuals with chronic diseases for management of disease and to gain insight on prognosis [4, 5], for example, in cardiovascular diseases [6, 7], respiratory disease [8], and cancer [9]. It is also used in risk–benefit evaluation of interventions [10–12] and for determining alternative care pathways such as rehabilitation before surgery for frail individuals [13, 14]. Meta-analyses of previous studies show the FFP to be associated with a higher risk of mortality in older community-dwelling individuals [15, 16].

A previous umbrella review on 25 meta-analyses concluded that frailty is associated with higher mortality risk, and the underlying pathological risk factors, i.e. chronic disease, may exacerbate the impact of frailty in different populations [17]. Whether the type of chronic disease plays a role in exacerbating the frailty-mortality association remains unknown. This is particularly true in middle-aged individuals where both chronic diseases and multimorbidity [18] and frailty [19] are increasingly prevalent. Better understanding of these associations would allow preventive measures directed at the management of chronic diseases and improvement in frailty status in middle-aged adults. To gain insight into the role of frailty, the overarching aim of our study is to examine the association of frailty status with mortality in analyses stratified by chronic diseases pertaining to 13 body organ systems. Data on 230,960 participants of the UK Biobank cohort study aged 38.0 to 72.0 years at inclusion, with one or more of these chronic diseases, were used to allow better understanding of the role of frailty in those with chronic diseases in mid-life and early old age. The secondary objective was to examine the frailty-mortality link in those with chronic diseases affecting multiple body organ systems.

## Methods

### Study population

The UK Biobank is a prospective, population-based cohort study that includes over 500,000 individuals, aged 40–69 years at recruitment between 2006 and 2010.

For the present study, we included participants from the baseline wave of the study who had at least one chronic disease, excluding participants free of chronic diseases. All participants provided written, informed consent for data collection, analysis, and access to their health records. UK Biobank received approval from the National Information Governance Board for Health and Social Care and the NHS North West Centre for Research Ethics Committee (reference number 11/NW/0382). This research was conducted using the UK Biobank Resource under data sharing application number 96856.

### Chronic diseases pertaining to 13 body organ systems

The UK Biobank study uses a system for ascertainment of chronic diseases using several electronic health records (primary care, hospital inpatient admissions, cancer registration using the unique National Health Service (NHS) identifier that is provided to all residents in the UK), and self-reported medical conditions using ICD-10 codes (International Classification of Diseases and Related Health Problems) [20]. For our analyses, we chose to exclude all self-reported chronic diseases that could not be confirmed in electronic health records.

A total of 47 chronic diseases were identified, and to produce results that were meaningful, we chose to group these diseases using the chapter classification of ICD-10. This classification is recommended by the World Health Organization (WHO) to allow grouping related diseases in chapters. We mapped the 47 chronic diseases to 13 body organ systems using ICD-10 classification chapters; excluded were chapters X11 and chapters XV to XXII due to non-relevance to midlife or insufficient number of cases in the chapters. The following 13 body organ systems were included in our analyses: infectious diseases, neoplasms, diseases of the blood and blood-forming organs, and certain disorders involving the immune mechanism, endocrine, nutritional and metabolic diseases, mental and behavioural disorders, nervous system, eyes and adnexa, ear and mastoid process, circulatory system, respiratory system, digestive system, musculoskeletal system and connective tissue, and genitourinary system. Details on the chronic diseases and the corresponding ICD-10 chapters are shown in Table S1.

### Mortality

The UK Biobank receives death notifications on a regular basis through linkage to national death registries, again using the unique NHS identifier for each participant. We used data on all-cause mortality up to 30th November 2022.

### Frailty

Fried’s Frailty phenotype [3] was measured at baseline, using a measure adapted to UK Biobank data proposed by Jiang and colleagues [21] including the following measures:

- *Weight loss* was determined based on participants report of weight change in the previous year and was categorised as reporting weight loss and no loss (without change or weight gain).
- *Exhaustion* was based on reported tiredness over the past 2 weeks, with responses from not at all, over several days, more than half the days, or nearly every day. Participants who reported tiredness on more than half the days or nearly every day were classified as meeting the criterion for exhaustion.
- *Slow walking pace* was determined based on answer to the question “how would you describe your usual walking pace?” with responses being slow, average, or brisk. Participants who reported slow walking pace were considered to fulfil the criterion for slow walking pace.
- *Physical activity* was categorised into four levels based on activities in the last four weeks: none (no physical activity), low (light activities such as pruning or watering the lawn), medium (walking for pleasure or heavy activities such as weeding, carpentry, or digging), and high (strenuous sports). Participants who reported no or low activity were classified as meeting the criterion for low physical activity.
- *Grip strength* was measured using a hydraulic hand dynamometer on both arms and the mean of these values or the measure available was used to determine this criterion. The cut-offs to define low grip strength were based on sex and body mass index as defined in the FFP.

The five criteria were summed to obtain a score from 0 to 5, and participants were classified as frail (score ≥ 3), pre-frail (1 or 2), or robust (0). Participants with incomplete data on one or more criteria were excluded (*N* = 8447), except when missing data did not change their frailty classification (*N* = 2261).

### Covariates

Socio-demographic factors included age, sex, living alone (yes, no), ethnicity (white, non-white), and socioeconomic deprivation (deprived/not deprived, dichotomization based on the median Townsend deprivation index). Health behaviours included smoking (current, former, or never-smoker), weekly alcohol consumption based on units of alcohol per week categorised as none (0), moderate (1 to 14), and above recommendations (> 14). Fruit and vegetable consumption was estimated by summing the reported intake of fresh, cooked, and dry fruits and vegetables over the course of a week and then categorised as recommended levels (≥ 5 portions per day) and low (< 5 portions per day) consumption. Body mass index was calculated using measured height and weight and categorised as normal (< 25 kg/m^2^), overweight (≥ 25 to 30 kg/m^2^), and obesity (≥ 30 kg/m^2^). The number of body organ systems affected by chronic diseases was categorised as 1, 2, or ≥ 3 out of 13 in the analyses to measure multimorbidity severity.

### Statistical analysis

The analyses reported in this study are based on UK Biobank participants with at least one chronic disease, as well as data on frailty and all covariates. We first examined the association of frailty status with mortality over the follow-up using Cox proportional hazards regression, without use of the ICD-10 chapter classification. In these analyses, follow-up time was the time scale, and the start of follow-up was the date of clinical assessment at baseline. The analyses were adjusted for socio-demographic factors, health behaviours, and multimorbidity severity (chronic diseases pertaining to 1, 2, ≥ 3 body organ systems). The results were expressed as hazard ratio (HR) with 95% confidence interval (CI). We then examined the link between frailty and mortality in each of the 13 groups of chronic diseases defined by the ICD-10 chapters, also using Cox regression as described above. These analyses were only undertaken in chronic disease groups with a minimum of 30 cases of mortality in each of the comparison groups. As the chronic disease groups were not independent (due to participants being able to be in multiple chapters), a formal test to compare the HRs across the 13 groups of chronic diseases could not be undertaken, leading us to examine whether the HR for each group of chronic disease overlapped with the confidence interval of the HR for ‘any chronic disease’.

We then calculated the population attributable fraction (PAF) to estimate the impact of frailty and prefrailty or frailty on mortality in each of the chronic disease groups using Miettinen’s formula [22]. PAFs are based on the prevalence and HR and represent the hypothetical proportion of mortality that would be avoided if the exposures were removed.

In additional analyses, we examined whether associations of frailty status with mortality differed as a function of sex, age at baseline (above or below 65 years old), and multimorbidity status (1 unique body system or combination of diseases affecting more than 1 unique body organ system) using an interaction term between frailty status and sex, age, or multimorbidity status, respectively. This analysis was conducted in the total population used in our study and for each of the 13 body organ systems. Multimorbidity patterns in the study population were plotted if prevalent in at least 1% of the study population.

Sensitivity analysis was conducted to assess the robustness of the main results. Main models were further adjusted for the presence of cardiovascular disease (defined as reporting any disease in the ICD-10 Circulatory diseases chapter except in analysis on that chapter), depression (defined using F32, F33 ICD-10 codes, except in analyses on the ‘Mental and behavioural’ chapter), and polypharmacy (defined as 5 or more self-reported medications); each of these was examined separately.

All analyses were conducted using R software (version 4.3.2) [23]. Cox regression analysis was run using the survival (version 3.5–5.5) package. All tests were two-tailed, and a *p*-value ≤ 0.05 was considered statistically significant.

## Results

From 502,206 participants at baseline in the UK Biobank study, 303,154 participants had at least one chronic disease, and among these, 230,960 had data on frailty status and all covariates (flow chart in Figure S1 in supplementary data). The analytical sample included a total of 230,960 (125,126 (54.2%) women) participants with at least one chronic disease; the mean age at baseline was 57.8 (± 7.8) years (Table 1). At baseline, 5.2% (11,962) of participants were frail, 43.6% (100,758) were prefrail, and 51.2% (118,240) were robust. Over a mean (SD) follow-up of 13.3 (2.1) years, a total of 23,198 (10.0%) participants died. More than half the participants in our analyses (51.7%) had multimorbidity, defined as diseases affecting at least two body systems. A higher proportion of mortality was observed in frail (20.8%, *n* = 2493) participants, compared to prefrail (11.2%, *n* = 11,327) or robust (7.9%, *n* = 9378) participants.

**Table 1:** Population characteristics by frailty status^a^ at baseline (2006–2010)

	Robust ***N*** = 118,240	Prefrail ***N*** = 100,758	Frail ***N*** = 11,962	Total ***N*** = 230,960
Age, mean (SD)	57.6 (7.8)	58.0 (7.8)	58.2 (7.5)	57.8 (7.8)
Sex, women	59,266 (50.1)	58,067 (57.6)	7793 (65.1)	125,126 (54.2)
Deprivation	28,461 (24.1)	32,776 (32.5)	5874 (49.1)	67,111 (29.1)
Marital status, single	9768 (8.3)	11,514 (11.4)	2062 (17.2)	23,344 (10.1)
Smoking status				
Never smoked	64,451 (54.5)	52,720 (52.3)	5604 (46.8)	122,775 (53.2)
Former smoker	44,694 (37.8)	37,956 (37.7)	4421 (37.0)	87,071 (37.7)
Current smoker	9095 (7.7)	10,082 (10.0)	1937 (16.2)	21,114 (9.1)
Alcohol consumption				
None	22,722 (19.2)	28,926 (28.7)	5790 (48.4)	57,438 (24.9)
Moderate	45,602 (38.6)	38,286 (38.0)	3713 (31.0)	87,601 (37.9)
Above recommendations	49,916 (42.2)	33,546 (33.3)	2459 (20.6)	85,921 (37.2)
Low fruits/vegetables consumption	20,703 (17.5)	20,233 (20.1)	3374 (28.2)	44,310 (19.2)
BMI				
Normal	39,393 (33.3)	23,505 (23.3)	1766 (14.8)	64,664 (28.0)
Overweight	54,649 (46.2)	42,305 (42.0)	3626 (30.3)	100,580 (43.5)
Obesity	24,198 (20.5)	34,948 (34.7)	6570 (54.9)	65,716 (28.5)
Multimorbidity	54,083 (45.7)	56,366 (55.9)	9013 (75.3)	119,462 (51.7)
Mortality^b^	9378 (7.9)	11,327 (11.2)	2493 (20.8)	23,198 (10.0)

*BMI* Body Mass Index, *SD* standard deviation

Statistics are *n* (column %) unless specified otherwise. Deprivation is defined as living in area below the median of the Townsend score

^a^Frailty defined using Fried’s Frailty Phenotype (5 criteria: weight loss, exhaustion, slow gait speed, low physical activity, and weak grip strength) with frailty defined as those having ≥3/5, pre-frailty as 1 or 2/5, and robust as those with a score of 0/5 on these criteria

^b^All cause mortality up to the 2022/11/30, median (Q1, Q3) follow-up = 13.7 (13.0, 14.4) years

The most frequently impaired body systems were the ‘Circulatory system’ (*n* = 89,239, 38.6%), ‘Musculoskeletal system’ (*n* = 88,663, 38.4%), and ‘Respiratory system’ (*n* = 49,822, 21.6%) (Table 2). Prevalence of frailty in body systems ranged from 5.1% (‘Neoplasms system’) to 10.0% (‘Endocrine, nutritional and metabolic system’) compared to 5.2% in the total study population. Prevalence of prefrailty ranged from 42.5% (‘Neoplasm system’) to 50.6% (‘Endocrine Nutritional and Metabolic system’) compared to 43.6% overall.

**Table 2:** Frailty^a^ at baseline and mortality over the follow-up as a function of chronic diseases pertaining to body organ systems

	Baseline frailty status			Total	Mortality over the follow-up
	Robust *N* (row %)	Prefrail *N* (row %)	Frail *N* (row %)	*N* (column %)^b^	*N* (row %)
Any chronic disease	118,240 (51.2)	100,758 (43.6)	11,962 (5.2)	230,960 (100)	23,198 (10.0)
Chronic diseases pertaining to 13 body organ systems, derived from ICD-10 classification					
Infectious and parasitic diseases (chapter I)	348 (44.3)	371 (47.2)	67 (8.5)	786 (0.3)	116 (14.8)
Neoplasms (chapter II)	16,428 (52.4)	13,309 (42.5)	1599 (5.1)	31,336 (13.6)	5013 (16.0)
Blood and blood-forming organs (chapter III)	3858 (45.0)	3974 (46.3)	750 (8.7)	8582 (3.7)	892 (10.4)
Endocrine, nutritional and metabolic diseases (chapter IV)	11,219 (39.4)	14,414 (50.6)	2854 (10.0)	28,487 (12.3)	4013 (14.1)
Mental and behavioural disorders (chapter V)	12,798 (42.8)	14,543 (48.7)	2532 (8.5)	29,873 (12.9)	2723 (9.1)
Nervous system (chapter VI)	13,441 (43.6)	14,632 (47.5)	2732 (8.9)	30,805 (13.3)	3406 (11.1)
Eye and adnexa (chapter VII)	8038 (49.1)	7202 (44.0)	1136 (6.9)	16,376 (7.1)	2359 (14.4)
Ear and mastoid process (chapter VIII)	4636 (51.0)	3988 (43.8)	473 (5.2)	9097 (3.9)	639 (7.0)
Circulatory system (chapter IX)	40,650 (45.6)	42,071 (47.1)	6518 (7.3)	89,239 (38.6)	12,731 (14.3)
Respiratory system (chapter X)	24,472 (49.1)	22,042 (44.2)	3308 (6.6)	49,822 (21.6)	5016 (10.1)
Digestive system (chapter XI)	17,986 (46.9)	17,645 (46.0)	2743 (7.2)	38,374 (16.6)	3634 (9.5)
Musculoskeletal system and connective tissue (chapter XIII)	41,676 (47.0)	40,262 (45.4)	6725 (7.6)	88,663 (38.4)	8956 (10.1)
Genitourinary system (chapter XIV)	8870 (50.8)	7506 (43.0)	1097 (6.3)	17,473 (7.6)	2342 (13.4)
Multimorbidity^c^					
Diseases affecting a unique body organ system	64,157 (57.5)	44,392 (39.8)	2949 (2.6)	111,498 (48.3)	8599 (7.7)
Diseases affecting multiple body organ systems	54,083 (45.3)	56,366 (47.2)	9013 (7.6)	119,462 (51.7)	14,599 (12.2)

Numbers are *n* (row %)

^a^Frailty defined using Fried’s Frailty Phenotype (five criteria: weight loss, exhaustion, slow gait speed, low physical activity, and weak grip strength) with frailty defined as those having ≥ 3/5, pre-frailty as 1 or 2/5, and robust as those with a score of 0/5 on these criteria

^b^Percentages do not add up to 100% as the chronic disease categories are not exclusive, participants with several diseases contributing to different categories of chronic disease

^c^Multimorbidity defined as the presence of two or more body organ systems affected by chronic diseases

**Fig. 1:**
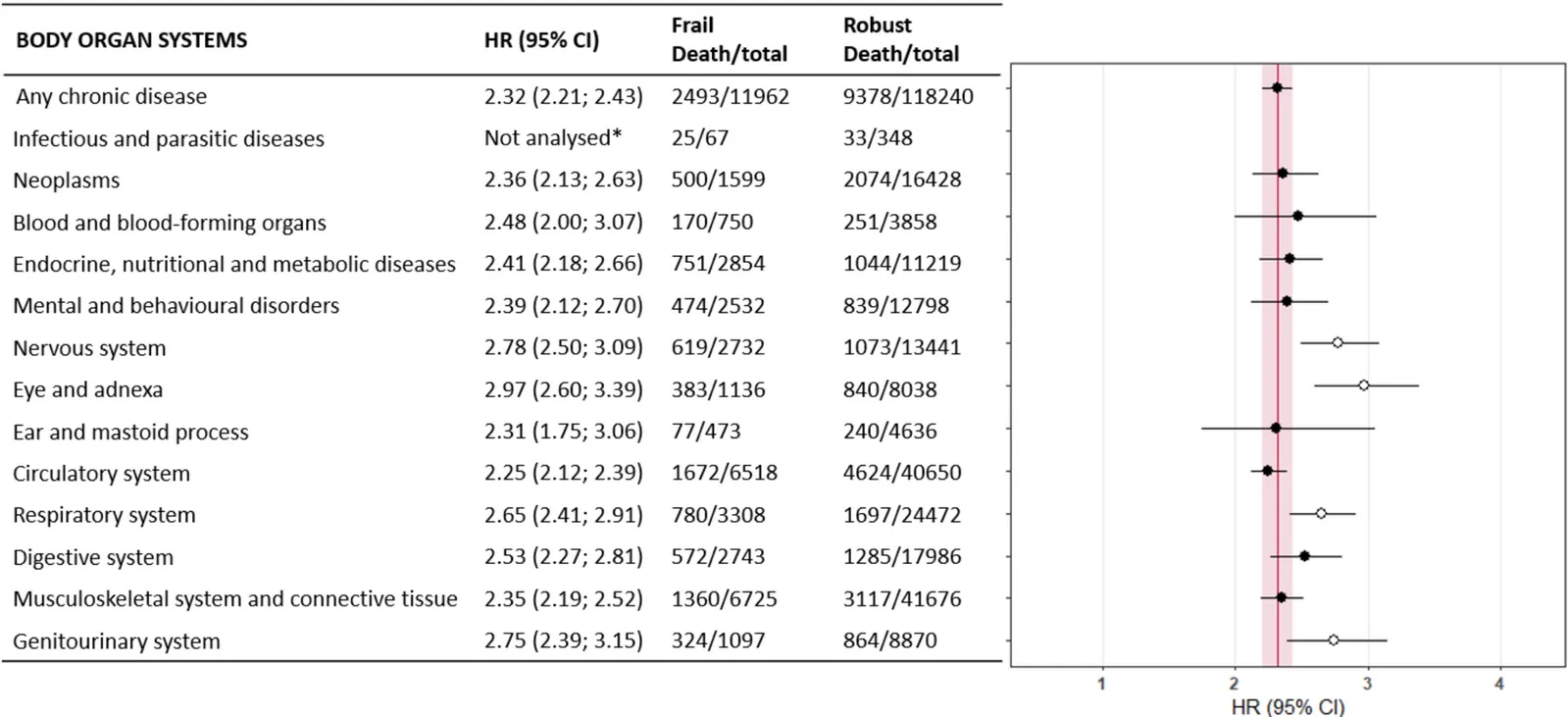
Hazard ratio of mortality for frailty vs robust over the total follow-up (13.3 (2.1) years) in individuals with unique or multiple chronic diseases pertaining to body organ systems. Estimates from Cox regression, adjusted for socio-demographic factors and health behaviors. The pink shaded area reflects the CI of the estimate for ‘any chronic disease’. Frailty phenotype (five criteria: weight loss, exhaustion, slow gait speed, low physical activity, and weak grip strength) with frailty defined as those having ≥ 3/5 and robust as those with a score of 0/5 on these criteria. The red solid line and shaded area show the reference estimate (association in those with any chronic disease) and 95% confidence intervals, respectively. *Not analysed as there were fewer than 30 cases of death in one of the comparison groups (frail or robust)

Figure 1 shows the risk of mortality in participants with frailty compared with those classified as being robust. In participants with any chronic disease, frailty was associated with a higher risk of mortality (HR 2.32; 95% CI 2.21, 2.43). In analyses of chronic diseases affecting specific body organ systems, this association ranged from 2.25 (2.12; 2.39) for ‘Circulatory system’ to 2.97 (2.60; 3.39) for ‘Eye and adnexa’. For four body systems, the confidence intervals of the HRs did not overlap with that of the associations for any chronic disease (HR greater than upper bound CI and lower bound CI did not include the HR for ‘any disease’). These were diseases of the ‘Nervous system’ (2.78 (2.50; 3.09)), ‘Eye and adnexa’ (2.97 (2.60; 3.39)), ‘Respiratory system’ (2.65 (2.41; 2.91)), and ‘Genitourinary system’ (2.75 (2.39; 3.15)), suggesting stronger associations between frailty and mortality in participants with chronic diseases relating to these chapters.

**Fig. 2:**
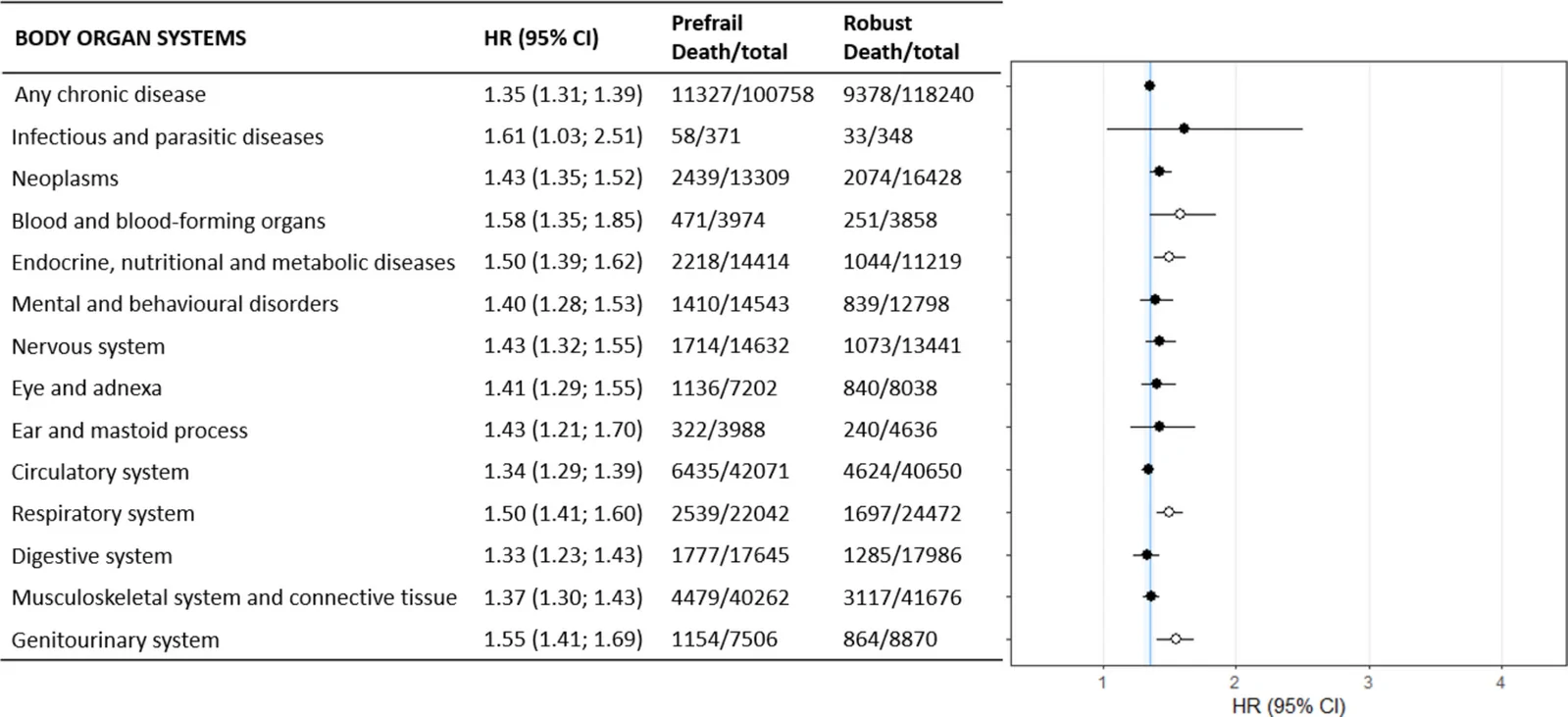
Hazard ratio of mortality for pre-frailty vs robust over the total follow-up (13.3 (2.1) years) in individuals with chronic diseases pertaining to body organ systems. *HR* Hazard Ratio, *CI* Confidence Interval. Blue shaded area reflects CI of the estimate for ‘any chronic disease’. Estimates from Cox regression, adjusted for socio-demographic factors, health behaviors, and multimorbidity severity (number of body organ systems affected by chronic diseases (1, 2, ≥3)). Fried’s Frailty Phenotype (5 criteria: weight loss, exhaustion, slow gait speed, low physical activity, and weak grip strength) with pre-frailty defined as 1 or 2/5, and robust as those with a score of 0/5 on these criteria. Blue solid line and shaded area show reference estimate (association in those with any chronic disease) and 95% confidence intervals, respectively

Figure 2 shows the risk of mortality in participants with prefrailty compared with those classified as being robust. For any chronic disease, the HR associated with prefrailty was 1.35 (1.31, 1.39). Associations ranged from 1.33 (1.23, 1.43) for ‘Digestive system’ to 1.61 (1.03, 2.51) for ‘Infectious system’. For four body systems, the prefrailty-mortality associations were greater than those observed in the total study population: ‘Blood system’ (1.58 (1.35, 1.85)), ‘Endocrine Nutritional and Metabolic system’ (1.50 (1.39, 1.62)), ‘Respiratory system’ (1.50 (1.41, 1.60)), and ‘Genitourinary system’ (1.55 (1.41, 1.69)).

Considering frailty status to be potentially modifiable, we calculated the PAF associated with frailty alone and then with prefrailty and frailty combined, results shown in Table 3. The PAF for frailty and in those with any chronic disease was 5.5% (5.1, 5.8) and ranged from 5.1% (4.4, 5.9) for ‘Neoplasms’ to 10.7% (9.3, 12.1) for ‘Eye and adnexa’. The PAF for prefrailty along with frailty in those with any chronic disease was 18.2% (17.0, 19.4), and the estimates for individual body organ systems ranged from 19.5% (17.7, 21.2) for diseases of the ‘Circulatory system’ to 34.0% (13.5, 48.5) for ‘Infectious and parasitic diseases’.

**Table 3:** Hazard ratios and population attributable fraction for the association of frailty status with mortality—analysis undertaken as a function of chronic diseases pertaining to body organ systems

	Frailty as compared to pre-frail/robust		Pre-frailty and frailty as compared to robust	
	HR (95% CI)	PAF% (95% CI)	HR (95% CI)	PAF% (95% CI)
Any chronic disease	1.92 (1.84; 2.00)	5.5 (5.1; 5.8)	1.43 (1.39; 1.47)	18.2 (17.0; 19.4)
Chronic diseases pertaining to 13 body organ systems, derived from ICD-10 classification				
Infectious and parasitic diseases (chapter I)	Not analysed^*^		1.87 (1.22; 2.86)	34.0 (13.5; 48.5)
Neoplasms (chapter II)	1.89 (1.71; 2.08)	5.1 (4.4; 5.9)	1.51 (1.42; 1.60)	20.1 (17.7; 22.5)
Blood and blood-forming organs (chapter III)	1.79 (1.50; 2.15)	9.1 (6.6; 11.5)	1.70 (1.45; 1.98)	29.9 (22.7; 36.2)
Endocrine, nutritional and metabolic diseases (chapter IV)	1.79 (1.65; 1.95)	9.0 (7.8; 10.1)	1.52 (1.51; 1.74)	28.7 (25.2; 32.1)
Mental and behavioural disorders (chapter V)	1.90 (1.71; 2.11)	8.6 (7.4; 9.9)	1.52 (1.40; 1.66)	23.9 (19.9; 27.7)
Nervous system (chapter VI)	2.20 (2.01; 2.41)	10.6 (9.5; 11.8)	1.60 (1.48; 1.72)	25.9 (22.5; 29.1)
Eye and adnexa (chapter VII)	2.37 (2.11; 2.66)	10.7 (9.3; 12.1)	1.57 (1.44; 1.72)	23.9 (20.0; 27.6)
Ear and mastoid process (chapter VIII)	1.83 (1.42; 2.37)	5.8 (3.7; 8.0)	1.52 (1.28; 1.79)	21.4 (13.8; 28.1)
Circulatory system (chapter IX)	1.86 (1.76; 1.97)	6.5 (6.0; 7.1)	1.43 (1.38; 1.49)	19.5 (17.7; 21.2)
Respiratory system (chapter X)	2.02 (1.86; 2.19)	8.5 (7.6; 9.4)	1.62 (1.52; 1.72)	25.6 (23.0; 28.2)
Digestive system (chapter XI)	2.10 (1.91; 2.31)	8.8 (7.8; 9.9)	1.46 (1.36; 1.57)	20.6 (17.3; 23.7)
Musculoskeletal system and connective tissue (chapter XIII)	1.92 (1.80; 2.04)	7.7 (7.0; 8.3)	1.48 (1.41; 1.55)	21.4 (19.3; 23.4)
Genitourinary system (chapter XIV)	2.09 (1.84; 2.37)	7.9 (6.7; 9.2)	1.67 (1.53; 1.82)	25.8 (22.2; 29.4)

*HR* Hazard Ratio, *CI* Confidence Interval, *PAF* Population Attributable Fraction

Estimates from Cox regression, adjusted for socio-demographic factors, health behaviours, and multimorbidity severity (number of body organ systems affected by chronic diseases (1, 2, ≥3))

*Not analysed as there were fewer than 30 cases of death in one of the comparison groups (Frail or Pre-frail/Robust)

Frailty Phenotype (5 criteria: weight loss, exhaustion, slow gait speed, low physical activity, and weak grip strength) with frailty defined as those having ≥3/5 (compared to those with <3/5, (Pre-frail/Robust)), and prefrailty/frailty as those with a score of ≥1/5 (compared to those with 0/5 (Robust)) on these criteria

Additional analyses suggested that frail (2.51 (2.36, 2.67)) and prefrail (1.38 (1.33, 1.43)) men with chronic diseases had a higher risk of mortality compared to women (2.09 (1.95, 2.24)) and (1.30 (1.24, 1.36)), respectively, *p* for interaction for frail < 0.01 and 0.03 for prefrail, Supplementary Figure S2 and S3. The frailty-mortality association was weaker in participants 65 years and older at baseline compared to those younger than 65 years, *p* for interaction < 0.01, Figure S4, but this was not the case for the prefrailty and mortality association, *p* for interaction = 0.46, Figure S5.

**Fig. 3:**
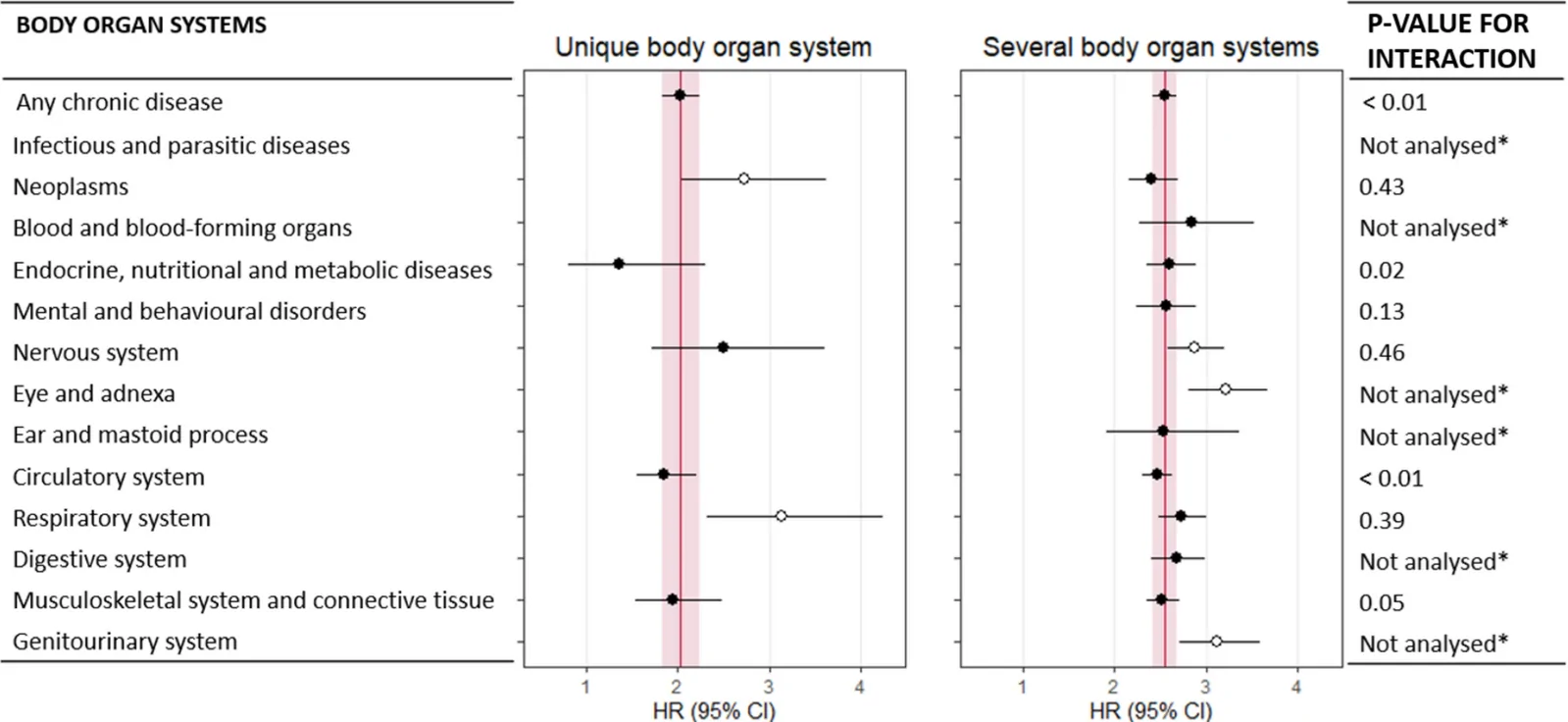
Hazard ratio of mortality for frailty vs robust over the total follow-up (13.3 (2.1) years) in individuals with unique or multiple chronic diseases pertaining to body organ systems. Estimates from Cox regression, adjusted for socio-demographic factors and health behaviors. Pink shaded area reflects CI of the estimate for ‘any chronic disease’. Frailty Phenotype (5 criteria: weight loss, exhaustion, slow gait speed, low physical activity, and weak grip strength) with frailty defined as those having ≥3/5, and robust as those with a score of 0/5 on these criteria. Red solid line and shaded area show reference estimate (association in those with any chronic disease) and 95% confidence intervals, respectively. *Not analysed as there were fewer than 30 cases of death in one of the comparison groups (Frail or Robust)

The multimorbidity patterns at the level of body organ systems, shown in Figure S6, suggest that diseases affecting the circulatory and/or musculoskeletal body organ systems were often present in multimorbidity combinations. Further analyses undertaken to compare the role of frailty in those with diseases in single and multiple body organ systems and frailty are shown in Fig. 3. In the total study population, the impact of frailty was greater in those with diseases affecting multiple (2.55 (2.42, 2.69)) compared to single body organ systems (2.03 (1.83, 2.24), *p* for interaction < 0.01). A similar pattern was observed for diseases of the endocrine (*p* for interaction = 0.02) and circulatory systems (*p* for interaction < 0.01). The association of prefrailty with mortality was also greater in participants with diseases in multiple (1.44 (1.39, 1.50)) compared to single system diseases (1.25 (1.20, 1.31)), *p* for interaction < 0.01, Figure S7. This effect was also evident for multimorbidity in those with diseases affecting blood (*p* for interaction = 0.01), circulatory (*p *for interaction < 0.01), and genitourinary (*p* for interaction = 0.04) systems.

Sensitivity analyses undertaken to examine the role of other covariates yielded results that were similar to those in the main analyses (Table S2).

## Discussion

This study on the importance of frailty for the risk of all-cause mortality in 230,960 participants with chronic diseases affecting 13 body organ systems presents four key findings. One, frailty was associated with a higher risk of mortality in all disease groups, with notably stronger associations observed for diseases relating to the nervous, eye, respiratory, and genitourinary systems. Two, PAF estimates suggested that, in those with any of the 13 categories of diseases, frailty and frailty/prefrailty could potentially account for 5.5% and 18.2% of deaths, respectively. The PAF estimates varied across diseases relating to the 13 body organ systems and were considerably higher for diseases of the nervous and eye systems. Three, the association between frailty and mortality was higher in participants with diseases affecting several body organ systems. Four, the frailty-mortality link was stronger in men than in women and in participants younger rather than older than 65 years at baseline. Taken together, these findings highlight the importance of the assessment of frailty in those with chronic diseases in midlife and early old age.

Previous studies have consistently shown a higher risk of mortality in frail individuals with chronic diseases, for example, in meta-analyses of studies on cardiovascular diseases [24], COPD [25], or cancer [26]. Much of the research in this domain is on older adults, as seen in the age at baseline of the studies included in these three meta-analyses being, for the vast majority, over 60 years old. Notable exceptions are studies focusing on specific populations such as adults living with HIV, adults with end-stage renal disease, or people who have specific surgeries such as organ transplant [27]. To our knowledge, our study is the first to examine the frailty-mortality link in mid-life to early old age as a function of chronic diseases relating to 13 body organ systems.

In our study population composed of people with chronic diseases, the prevalence of frailty was only 5.2%, comparable to 6% (4–8%) reported in the 50–59 + age group in a recent meta-analysis [28]. The prevalence of prefrailty in our study population was 43.6%, again comparable to 41% (38–44%) in the 50–59 + age group in the meta-analysis [28]. Remarkably, compared to robust participants, those with prefrailty also had a higher risk of mortality, irrespective of the chronic disease chapter considered in the analyses. The exposure–outcome associations were stronger for frailty than prefrailty, but there are at least two issues that need further reflection. One, the prevalence of prefrailty was higher, suggesting that the populational impact of prefrailty is higher. Two, it is possible that prefrailty may be more amenable to modification than frailty [29, 30], making it important to consider its role in individuals with chronic diseases.

Considering prefrailty and frailty together suggests that a third (infectious and parasitic diseases, blood and blood-forming organs, endocrine, nutritional and metabolic diseases) to a quarter (nervous system, respiratory system, genitourinary system) of death cases might be addressed by targeting frailty and prefrailty in those with chronic diseases pertaining to specific body organ systems. These results need to be considered in light of the fact that PAF estimates rely both on the prevalence of the exposure and the strength of the outcome association. The prevalence of prefrailty and frailty was highest in the “endocrine, nutritional and metabolic diseases” chapter, partly explaining the high PAF values. It is also possible that patterns of comorbidity across these chapters differ, leading to somewhat higher or lower PAF estimates. Another possibility is that certain diseases (e.g. eye diseases) have an impact on day-to-day function, increasing the risk of mortality. While the precise mechanisms require further research, our findings highlight the importance of frailty in those with chronic diseases, even in midlife.

There were some differences in associations as a function of sex and age at baseline. The prevalence of frailty was higher in women, as also reported in other studies [28, 31], but the associations of frailty with mortality were higher in men. This pattern has been reported previously and is thought to be due to greater physiological reserve in women or other biological and psychosocial factors [32]. Sex-related differences also exist in the diagnosis or management of several chronic diseases [33–35] and in frailty [36]. In relation to age, there is consistent evidence of an increase in frailty with age 4 [8], and it is already present in midlife [19, 28]. Our results show higher frailty in participants 65 years and older, but the frailty-mortality link was stronger in the younger participants, highlighting the relevance of frailty screening irrespective of their age. The measure of frailty and the thresholds for its components in the FFP were defined in participants 65 to 101 years [3], and middle-aged individuals meeting these criteria might be particularly compromised in physiological reserve. Whether frailty in midlife reflects early aging or a more severe form of frailty remains unclear.

Multimorbidity is increasingly common across the adult lifespan [18], and even with our strict definition (chronic disease affecting more than one of the 13 body organ systems rather than chronic diseases considered individually), it was seen in 51.7% of the study population. The associations of frailty and prefrailty with mortality in those with multimorbidity were systematically stronger than in persons affected by diseases within a single body organ system. A previous study conducted in 493,737 participants of the UK Biobank also found a higher risk of death among frail multimorbid participants compared to those who were non-frail and multimorbid using the conventional definition of multimorbidity (two or more diseases, irrespective of affected body organ systems) [19]; the HR estimates ranged from 2.49 (women aged 55–65 years) to 3.89 (men aged 37–45 years). However, the focus of the study was not participants with chronic diseases, and they used individual chronic diseases rather than multimorbidity defined across body organ systems. A recent study examined frailty transitions in 7675 multimorbid participants aged 65 and over, differentiated into 4 clusters of multimorbidity [37]. The results showed a higher risk of transition from prefrailty or frailty to mortality in participants with a higher multimorbidity count and for specific multimorbidity patterns. Taken together, these results suggest that, irrespective of the definition of multimorbidity, stronger associations of frailty with mortality are seen in multimorbid individuals.

The mechanisms underlying the frailty-mortality association remain to be elucidated, but several studies show frailty to be associated with inflammatory biomarkers [38] that are thought to play a role in the disruption of key biological features of ageing [39, 40]. There is also evidence indicating that several markers of accelerated biological ageing are closely associated with the onset of frailty and risk of mortality [41]. At the organ level, abnormalities such as cardiac and vascular alterations, impaired lung function, anaemia, and renal dysfunction are also more likely to be observed in frail compared to robust individuals, whether or not they have a diagnosed disease [42]. In addition, frailty has been identified as a prognostic marker for a number of diseases, including cardiovascular disease [43, 44], COPD [45] and rheumatoid arthritis [46]. Taken together, these findings suggest that frailty may not simply accompany poorer biological and organ function but may also reflect greater disease severity across a broad range of clinical conditions.

Frailty screening is recommended for older adults with chronic diseases [47], and our results clearly show the importance of frailty screening in persons with chronic diseases, irrespective of the age of the person and the type of chronic disease. Adapting healthcare pathways to include assessment of frailty status is likely to be important as frailty is itself associated with adverse health outcomes (disability, morbidity, institutionalisation, and death), and there is an urgent need to integrate frailty into medical specialties, beyond geriatrics [48]. Interventions for reversal or reduction of pre-frailty and frailty in adults 65 years involving group physical exercise, protein supplementation, and cognitive training have been shown to be effective [49]. Whether this strategy works in middle-aged adults is unclear as a recent review of interventions in adults 40 to 65 years identified significant evidence gaps and limitations in methodology [50].

A major strength of our study lies in the substantial sample size, allowing the investigation of frailty phenotype in participants with chronic diseases across several body organ systems. Comprehensive linkage to multiple electronic databases and national registers ensures the validity of the diagnostic categories compared to self-reported data. The findings of our study need to be considered in light of several limitations. One, the prevalence of diseases affecting certain body organ systems (infectious diseases system, blood system, eye diseases, etc.) did not allow analyses on some groups and multimorbidity patterns. Due to the high prevalence of multimorbidity, we chose not to exclude participants with other chronic diseases in the analyses of individual chronic disease chapters, implying that the estimates for a particular chapter cannot solely be attributed to that chapter. Two, UK Biobank participants are generally healthier than the UK general population [21] and may not be representative of the general population. This may not necessarily affect the observed exposure-outcome associations [21], but the prevalence of frailty may be lower, and the resulting PAFs may have been underestimated. Three, the measure of frailty used in our analyses is an adapted version of the FFP and may not completely reflect the original measure. However, this measure has been used in several studies using UK Biobank data and is unlikely to be a major source of bias. Four, the use of PAFs is useful as a summary measure, but it does assume that frailty and prefrailty have “causal” associations with mortality and can be removed effectively.

In conclusion, our findings highlight the importance of screening for frailty in individuals with chronic diseases, particularly diseases of the respiratory, genitourinary, nervous, and eye systems, as well as in the presence of diseases affecting multiple body organ systems. Further research is needed to examine the effectiveness and cost-efficiency of interventions targeting frailty and prefrailty in those with chronic diseases in midlife, in multiple settings, for reduction in the risk of mortality.

## Supplementary information

Below is the link to the electronic supplementary material.

ESM 1 (DOCX 927 KB)

## Data Availability

UKB data is available, upon request, by following the application procedure detailed in https://www.ukbiobank.ac.uk/use-our-data/apply-for-access. This work uses data provided by patients and collected by the NHS as part of their care and support.
