# Supplementary material for: The frailty mortality link in people with chronic diseases pertaining to 13 body organ systems: findings from the UK Biobank study
Source: GeroScience. 2025 Dec 6;48(5):6915–27. doi: 10.1007/s11357-025-01980-4 (PMC13601399; doi:10.1007/s11357-025-01980-4)
Supplement: Supplementary file 1 [file 11357_2025_1980_MOESM1_ESM.docx]

**Supplementary data**

**Table S1. Chronic diseases pertaining to 13 body organ systems used in the analyses.**

| **Body organ system** | **Disease group** | **ICD-10 code** |
| --- | --- | --- |
| Infectious and parasitic diseases  (chapter I) | Tuberculosis | A15; A16; A17; A18; A19 |
|  | Chronic hepatitis | B18 |
|  | HIV | B20; B21; B22; B23; B24 |
| Neoplasms (chapter II) | All listed cancers | / |
| Diseases of the blood and blood-forming organs and certain disorders involving the immune mechanism (chapter III) | Nutritional anaemias | D50; D51; D52; D53 |
|  | Haemolytic anaemias | D55; D56; D57; D58; D59 |
|  | Anaemia in chronic diseases | D63 |
| Endocrine, nutritional and metabolic diseases (chapter IV) | Disorders of thyroid gland | E01; E03; E04; E05 |
|  | Diabetes | E10; E11; E14 |
| Mental and behavioural disorders (chapter V) | Mood disorders | F31; F32; F33 |
|  | Neurotic stress | F40; F41; F43 |
| Diseases of the nervous system  (chapter VI) | Systemic atrophies primarily affecting the central nervous system | G10; G11; G12; G13; G14 |
|  | Parkinsonisms | G20; G21; G22; G23 |
|  | Other degenerative diseases of the nervous system | G30; G31; G32 |
|  | Multiple sclerosis | G35 |
|  | Episodic and paroxysmal disorders | G40; G43; G45; G46; G47 |
|  | Mononeuropathies | G56; G57 |
|  | Cerebral palsy and other paralytic syndromes | G80; G81; G82; G83 |
| Diseases of the eye and adnexa  (chapter VII) | Cataract | H25; H26 |
|  | Disorders of choroid and retina | H30; H31; H32; H33; H34; H35; H36 |
|  | Glaucoma | H40 |
| Diseases of the ear and mastoid process (chapter VIII) | Diseases of inner ear | H80; H81; H82; H83 |
| Diseases of the circulatory system (chapter IX) | Hypertensive diseases | I10; I11; I12; I13; I15 |
|  | Ischemic heart diseases | I20; I21; I22; I23 |
|  | Valves diseases | I34; I35; I37 |
|  | Heart failure | I50 |
|  | Arrythmia | I44; I45; I47; I49 |
|  | Stroke | I60; I61; I63; I64 |
|  | Aneurysms | I71; I72 |
|  | Chronic rheumatic heart disease | I05; I06; I07; I08; I09 |
| Diseases of the respiratory system (chapter X) | Vasomotor and allergic rhinitis | J30 |
|  | Chronic rhinitis, nasopharyngitis and pharyngitis | J31 |
|  | Chronic sinusitis | J32 |
|  | Chronic laryngitis | J37 |
|  | Chronic bronchitis | J40; J41; J42 |
|  | Chronic obstructive pulmonary disease | J43; J44 |
|  | Asthma | J45; J46 |
|  | Bronchiectasis | J47 |
| Diseases of the digestive system  (chapter XI) | Gastro-oesophageal reflux diseases | K21; K22 |
|  | Functional intestinal disorders | K58; K59 |
| Diseases of the musculoskeletal system and connective tissue (chapter XIII) | Inflammatory polyarthropathies | M05; M06; M07; M09; M10; M11; M12; M13; M14 |
|  | Arthrosis | M15; M16; M17; M18; M19 |
|  | Dorsopathies | M40; M41; M42; M43; M45; M46; M47; M48; M49; M50; M51; M53; M54 |
|  | Shoulder lesions | M75 |
|  | Osteoporosis | M80; M81 |
| Diseases of the genitourinary system (chapter XIV) | Chronic kidney disease | N18 |
|  | Endometriosis | N80 |
|  | Hyperplasia of prostate | N40 |

Abbreviations: ICD-10: International Classification of Diseases and Related Health Problems, version 10.

**Table S2:** **Association of frailty and pre-frailty with mortality in sensitivity analysis in models further adjusted for the presence of cardiovascular diseases (defined as reporting any disease in the ICD-10 Circulatory diseases chapter), depression (defined using F32, F33 ICD-10 codes) or polypharmacy (defined as 5 or more self-reported medication).**

| **Body Organ system** | **Adjusted for cardiovascular diseases** | | **Adjusted for depression** | | **Adjusted for polypharmacy** | |
| --- | --- | --- | --- | --- | --- | --- |
|  | Frailty  HR (95% CI) | Pre-frailty  HR (95% CI) | Frailty  HR (95% CI) | Pre-frailty  HR (95% CI) | Frailty  HR (95% CI) | Pre-frailty  HR (95% CI) |
| **Any chronic disease** | 2.24 (2.11; 2.38) | 1.34 (1.29; 1.39) | 2.31 (2.20; 2.42) | 1.35 (1.31; 1.39) | 2.10 (2.00; 2.20) | 1.31 (1.27; 1.35) |
| **Infectious and parasitic diseases** | *Not analysed | 1.57 (0.80; 3.09) | *Not analysed | 1.61 (1.03; 2.51) | *Not analysed | 1.61 (1.03; 2.51) |
| **Neoplasms** | 2.40 (2.07; 2.79) | 1.51 (1.37; 1.67) | 2.37 (2.13; 2.63) | 1.43 (1.35; 1.52) | 2.15 (1.93; 2.39) | 1.40 (1.31; 1.48) |
| **Blood and blood-forming organs** | 2.97 (2.22; 3.96) | 1.80 (1.41; 2.29) | 2.47 (1.99; 3.06) | 1.58 (1.35; 1.85) | 2.14 (1.72; 2.66) | 1.50 (1.28; 1.76) |
| **Endocrine, nutritional and metabolic diseases** | 2.29 (2.03; 2.59) | 1.49 (1.35; 1.64) | 2.40 (2.17; 2.66) | 1.50 (1.39; 1.62) | 2.19 (1.34; 1.56) | 1.45 (1.34; 1.56) |
| **Mental and behavioural disorders** | 2.38 (2.01; 2.81) | 1.44 (1.26; 1.65) | *Not analysed | *Not analysed | 2.07 (1.83; 2.35) | 1.34 (1.23; 1.47) |
| **Nervous system** | 2.55 (2.22; 2.94) | 1.34 (1.19; 1.49) | 2.76 (2.48; 3.07) | 1.43 (1.32; 1.54) | 2.41 (2.16; 2.68) | 1.35 (1.25; 1.47) |
| **Eye and adnexa** | 2.95 (2.50; 3.48) | 1.40 (1.24; 1.59) | 2.97 (2.60; 3.39) | 1.41 (1.29; 1.55) | 2.59 (2.26; 2.96) | 1.35 (1.23; 1.48) |
| **Ear and mastoid process** | 2.52 (1.75; 3.63) | 1.52 (1.17; 1.98) | 2.30 (1.74; 3.05) | 1.43 (1.21; 1.70) | 2.08 (1.56; 2.77) | 1.39 (1.17; 1.65) |
| **Circulatory system** | *Not analysed | *Not analysed | 2.24 (2.11; 2.38) | 1.34 (1.29; 1.39) | 2.07 (1.95; 2.20) | 1.30 (1.25; 1.35) |
| **Respiratory system** | 2.73 (3.40; 3.10) | 1.66 (1.51; 1.84) | 2.62 (2.39; 2.89) | 1.49 (1.40; 1.59) | 2.36 (2.15; 2.59) | 1.44 (1.35; 1.54) |
| **Digestive system** | 2.28 (1.99; 2.62) | 1.28 (1.15; 1.42) | 2.52 (2.26; 2.80) | 1.33 (1.23; 1.43) | 2.28 (2.05; 2.54) | 1.29 (1.20; 1.39) |
| **Musculoskeletal system and connective tissue** | 2.33 (2.13; 2.55) | 1.39 (1.30; 1.49) | 2.35 (2.19; 2.51) | 1.37 (1.30; 1.43) | 2.11 (1.97; 2.27) | 1.32 (1.26; 1.38) |
| **Genitourinary system** | 2.70 (2.30; 3.19) | 1.57 (1.40; 1.77) | 2.73 (2.38; 3.14) | 1.55 (1.41; 1.69) | 2.40 (2.08; 2.76) | 1.48 (1.35; 1.62) |

Abbreviation: HR: Hazard Ratio, CI: Confidence Interval. Pink shaded area reflects CI of the estimate for ‘any chronic disease’.

Estimates from Cox regression, adjusted for socio-demographic factors, health behaviors, and multimorbidity severity (number of body organ systems affected by chronic diseases (1, 2, ≥3)) and further for cardiovascular diseases, depression or polypharmacy.

Frailty Phenotype (5 criteria: weight loss, exhaustion, slow gait speed, low physical activity, and weak grip strength) with frailty defined as those having ≥3/5, pre-frailty as 1 or 2/5, and robust as those with a score of 0/5 on these criteria (used as reference).

*Not analysed as there were fewer than 30 cases of death in one of the comparison groups (Frail, Prefrail or Robust) and if adjusted for the same body organ system.

**Figure S1. Flow chart of the study population.**

**Figure S2. Hazard ratio of mortality for frailty vs robust over the total follow-up (13.3 (2.1) years) in individuals with chronic diseases pertaining to body organ systems in men and in women**

Abbreviations: HR: Hazard Ratio; CI: Confidence Interval. Pink shaded area reflects CI of the estimate for ‘any chronic disease’.

Estimates from Cox regression, adjusted for socio-demographic factors, health behaviors, and multimorbidity severity (number of body organ systems affected by chronic diseases (1, 2, ≥3)).

Frailty defined using Fried’s Frailty Phenotype (5 criteria: weight loss, exhaustion, slow gait speed, low physical activity, and weak grip strength) with frailty defined as those having ≥3/5, and robust as those with a score of 0/5 on these criteria. Red solid line and shaded area show reference estimate (association in those with any chronic disease) and 95% confidence intervals, respectively.

*Not analysed as there were fewer than 30 cases of death in one of the comparison groups (Frail or Robust).

**Figure S3. Hazard ratio of mortality for prefrailty vs robust over the total follow-up (13.3 (2.1) years) in individuals with chronic diseases pertaining to body organ systems in men and in women**

Abbreviations: HR: Hazard Ratio; CI: Confidence Interval. Blue shaded area reflects CI of the estimate for ‘any chronic disease’.

Estimates from Cox regression, adjusted for socio-demographic factors, health behaviors, and multimorbidity severity (number of body organ systems affected by chronic diseases (1, 2, ≥3)).

Fried’s Frailty Phenotype (5 criteria: weight loss, exhaustion, slow gait speed, low physical activity, and weak grip strength) with pre-frailty defined as 1 or 2/5, and robust as those with a score of 0/5 on these criteria. Blue solid line and shaded area show reference estimate (association in those with any chronic disease) and 95% confidence intervals, respectively.

*Not analysed as there were fewer than 30 cases of death in one of the comparison groups (Prefrail or Robust).

**Figure S4. Hazard ratio of mortality for frailty vs robust over the total follow-up (13.3 (2.1) years) in individuals with chronic diseases pertaining to body organ systems in analyses stratified by age at baseline**

Abbreviations: HR: Hazard Ratio; CI: Confidence Interval. Pink shaded area reflects CI of the estimate for ‘any chronic disease’.

Estimates from Cox regression, adjusted for socio-demographic factors, health behaviors, and multimorbidity severity (number of body organ systems affected by chronic diseases (1, 2, ≥3)).

Frailty defined using Fried’s Frailty Phenotype (5 criteria: weight loss, exhaustion, slow gait speed, low physical activity, and weak grip strength) with frailty defined as those having ≥3/5 and robust as those with a score of 0/5 on these criteria. Red solid line and shaded area show reference estimate (association in those with any chronic disease) and 95% confidence intervals, respectively.

*Not analysed as there were fewer than 30 cases of death in one of the comparison groups (Frail or Robust).

**Figure S5. Hazard ratio of mortality for prefrailty vs robust over the total follow-up (13.3 (2.1) years) in individuals with chronic diseases pertaining to body organ systems in analyses stratified by age at baseline**

Abbreviations: HR: Hazard Ratio; CI: Confidence Interval. Blue shaded area reflects CI of the estimate for ‘any chronic disease’.

Estimates from Cox regression, adjusted for socio-demographic factors, health behaviors, and multimorbidity severity (number of body organ systems affected by chronic diseases (1, 2, ≥3)).

Fried’s Frailty Phenotype (5 criteria: weight loss, exhaustion, slow gait speed, low physical activity, and weak grip strength) with pre-frailty defined as 1 or 2/5, and robust as those with a score of 0/5 on these criteria. Blue solid line and shaded area show reference estimate (association in those with any chronic disease) and 95% confidence intervals, respectively.

*Not analysed as there were fewer than 30 cases of death in one of the comparison groups (Prefrail or Robust).

**Figure S6. Distribution of multimorbidity patterns**

**Figure S7. Hazard ratio of mortality for prefrailty vs robust in those with one and multiple chronic diseases pertaining to body organ systems**

Estimates from Cox regression, adjusted for socio-demographic factors, and health behaviors. Blue shaded area reflects CI of the estimate for ‘any chronic disease’.

Fried’s Frailty Phenotype (5 criteria: weight loss, exhaustion, slow gait speed, low physical activity, and weak grip strength) with pre-frailty defined as 1 or 2/5, and robust as those with a score of 0/5 on these criteria. Blue solid line and shaded area show reference estimate (association in those with any chronic disease) and 95% confidence intervals, respectively.

*Not analysed as there were fewer than 30 cases of death in one of the comparison groups (Prefrail or Robust).
